# Supplementary material for: Patterns of Intron Gain and Loss in Fungi
Source: PLoS Biol. 2004 Nov 30;2(12):e422. doi: 10.1371/journal.pbio.0020422 (PMC532390; doi:10.1371/journal.pbio.0020422)
Supplement: Table S1 — Also available at http://genes.mit.edu/NielsenEtAl/. (4.3 MB ZIP). [file pbio.0020422.st001.zip › NielsenEtAl/html/700.html]

AN4759.1.NCU01911.1.MG02923.1.FG08936.1


```
 CLUSTAL W (1.82) Multiple Sequence Alignments - Introns Inserted


Sequence 1: NCU01911.1	793 aa
Sequence 2: FG08936.1	675 aa
Sequence 3: MG02923.1	669 aa
Sequence 4: AN4759.1	605 aa
Alignment Length: 800 aa
Number Identitical Residues: 166 aa
Alignment Score (without introns) 9074


MG02923.1 	---------~-------------------------MSADVAYASTFLGPAHN--------
NCU01911.1	MAAVSTTNG~LWQSFVTVTGWSSHQIPIPANGRLSKSHTRSLSSILSRNSASRASISDAT
FG08936.1 	------MDS2FIPMSAAAAGWGQPLATKSPASRRSLGHFRSLSSIAAAPSYH--------
AN4759.1  	------MAD2LIAFHSYYS--HQGFLSPSNMLPRPSSHKRSLSSGNRSASPD--------
          	        .         :   .   . .     . .   : :*     :          

MG02923.1 	--------SKTPSPTSATPRN--------FDDSQDLNTLPDPRSRAMSPASAHGFPSPIS
NCU01911.1	PELPSVSEPASANAAPSTPRLRRPFSPQPIDDENELTTLPDPRSRAMSPASGVSMTG--H
FG08936.1 	PRTPSLTPDRTPIKSSSSTHLPLGVKSASVDFGDEMSTIPDPRSRAISPADDSGVATP-N
AN4759.1  	---RHVTKAKSTNDLLRVAADPPATVRSSSEGGDGFSTLRDPRLLPSSESTESTLSS--S
          	      :   :.      .         . :  : :.*: ***  . * :    ..    

MG02923.1 	PVSDLDLEVATLSTKLINAINHQTTLDDTLSRARAELEDARETIRQLEGRLAEQREMLSG
NCU01911.1	HHPDLDQEVATLSTKLINAINHQTTLDDNLSQTKMELEQARDKIRQLERTVEEQREMLAG
FG08936.1 	HHPDLNDEVATLSNKLINAINHQTILDDSLSAARHELDAARDRIRDLETQNASQREMLAG
AN4759.1  	HHPDLNDEVATLSLKLVQAINNQTKLDDTLAVTRQELEQVQEKARALESENEQYWRDIED
          	  .**: ****** **::***:** ***.*: :: **: .::  * **    .  . : .

MG02923.1 	DVWVRRKTVESEKKDLLRRVADEKKARYEMEHQKKKIEQELEELSTQLFEEAQK~MVIAA
NCU01911.1	DVWVRRKTVEAEKTTLLGVIAEEKQARLDVEQQKKKIEQELENLTAALFEEANK~MVISA
FG08936.1 	DVWVRKHTVEAEKKVWQAKIAVERQKRLDTEMEKKKIEQELENLTAALFEEANK~MVIAA
AN4759.1  	GALVKKAEVDQLK----ASLAEEKAQRLLAEKEKKGIEQELETLTAALFEEANK0MVAAA
          	.. *::  *:  *      :* *:  *   * :** ****** *:: *****:* ** :*

MG02923.1 	KEDAQKEQEILHNKNEKLRDQLSNTEILLKLQNDQLAELKAVMEQMHVDREDQNALTAPS
NCU01911.1	KEEARIEQEILQRKNDQLRAQLVDTEGLLKSQQEQLAELKHLMEDMSAEKHEQIPPTAPS
FG08936.1 	KEEAKADHEVLQRKNDQLKSQLADTESILKSQQEQLVELKHVMEHMAAGREEHTNPTAPS
AN4759.1  	KLER----EAVEKKNEQLRSQIKDTESLLASHQEQLAELKSVLQVMNISKDDLDGRTAIS
          	* :     * :..**::*: *: :** :*  :::**.*** ::: *   :.:    ** *

MG02923.1 	SPGFSRFDSKDDETPFS-------AADESLPEPVSPSYPTSFTHLVQPILRTDLSSYEDF
NCU01911.1	SPGFSKFELQNEEGLSDRLHHGHHDRQLSVAESMSPSYPTSFTYLLKPVLRTDLASYNDF
FG08936.1 	SPGLTKPDTRDDRRSST---EEPMPSLWGAAAVVEPAHPTSFSHLIQPVLRTDLASYDDF
AN4759.1  	TAPPSPAGPSQPPAFTRKSMEATDIPGDVVCAEIAPGPATSFPDLIKTVCRTDILAYEDF
          	:.  :     :                      : *. .***. *::.: ***: :*:**

MG02923.1 	KILVQTTKRLSVRSRPPSGAFGAS-AMSALGLT-AGHSMSPSNASTSSVASTVPTSSSGS
NCU01911.1	KDLIRTSKRISAQQRVPTPSTGSALTSLGLGLGSVGAHLALANASSTSLSTIATAQ--ST
FG08936.1 	VSLTRIS-RNRASSRVSSGSVSALNALTGFGLGGSISSAHPSNASTTSLGTPIPGP--GS
AN4759.1  	RELFTISKSSKPPSRATSGSYSGLNVMSLAGFGGAGFGSGSPSPSKS-------------
          	  *   :.     .* .: : ..  .    *: .       ...*.:             

MG02923.1 	LPQTPNTPGSAALSAGSGAAAVPVPPLKDTRFFKRVLSEDIEPTLRLDIAPGLSWLSRRT
NCU01911.1	FSQTSTTP-TTAVTASPGAAAIPLPALKDTRFYKRVLTEDIEPTLRLDIAPGLSWLARRS
FG08936.1 	APQSPNTP-ASTVSAASNASATPLPSLRETKFYKRVLAEDIEPTLRLDMAPGLSWLARRS
AN4759.1  	---QPHSPSGSVSSPQPPNSSVP---LKDARFYKRALVEDIEPTLRLDAAPGISWLTRRN
          	    . :*. :. :. .  :: *   *::::*:**.* ********** ***:***:**.

MG02923.1 	VINAITEGTLVVEPVPP--ATPGSLVAITKPQFKECSLCGENRKEEAHLRTYRFRTSDAD
NCU01911.1	VLTAMTDGSLVVEPVPAATTTTSRFGRVSRPELSPCSLCGESRPEEEFLRKHRFRISETG
FG08936.1 	VIASIADGTLVVEPVP----TTGSLVALTKPQFYPCSLCGDSRKGPEYLRNHRFRTSETD
AN4759.1  	VLSSICEGSLVVEPVPP---------NVRKYEF-PCSLCGERRSGTDNERTHRFRTSDSE
          	*: :: :*:*******.          : : ::  *****: *      *.:*** *:: 

MG02923.1 	TAQR-YPLCKSYCLVRVRATCDLMGFLRLLKDGHWRTDDEDAVKAAWEESVRLREQMFWA
NCU01911.1	SAQTGYALCR-YCLSRVRSTCDFLGFLRIVKDGHWRADDEDAEKAAWAESVRLREQMFWS
FG08936.1 	SAQR-HPLCK-YCLSRVRSTCDFLGFLRMVKDGHWRADNEDHEKAAWEESVRLREQMFWS
AN4759.1  	TAQR-YPLCV-LCLEKVRSCCEFAGYLRLILDGHIHAEEPDELQEAWEETVRLRERMFWS
          	:**  :.**   ** :**: *:: *:**:: *** :::: *  : ** *:*****:***:

MG02923.1 	RIGGGVVPTTPMLIGKSPMLPEKSPRPSQDGKNEAPQVDQQQSQEPPKPSVEVSSESSDN
NCU01911.1	RIGGGVLPITHFHTSHSRAPSRAPSLRGDKSSRTSLETDKDGDETPAAGEVEQLAVSVAE
FG08936.1 	RIGGGVIPSIPAPISIDMEKS---PRNSDDGSVRSDHLEVPGFQTPPT----TMERTLSD
AN4759.1  	RIGGGIVPTFAQTSAPE-------ERSEGHQASIDSLIEKPVVQIQPS-----DDIQDTA
          	*****::*      . .            .        :    :  .             

MG02923.1 	STAPAPAGAPDTKPEGLGLQNVS-------------------------------------
NCU01911.1	VADAIQQESSETEIPEVLEQLTSNIATEPVDQPEITQKDEAPVEEVVVPIAISEVSEEQV
FG08936.1 	ISNAIP------------------------------------------------------
AN4759.1  	IG----------------------------------------------------------
          	                                                            

MG02923.1 	------------------------LTSPTSETVEEKVARPVTPPPKVSEEQMPPASNTNE
NCU01911.1	EIIEQKVEAPTDDMEGPTEETKLVWEEPKTPANKFEFLEAEITQCPPSTEDTPKQPVASE
FG08936.1 	------------------------IEEPHTPPMQID---------GPRSVRTSVQSLEVK
AN4759.1  	------------------------------------------------QEKLTATPRPLA
          	                                                    .  .    

MG02923.1 	ADADADGANKQTTPQIPAQQNQ
NCU01911.1	VEPATE-EPKELAVTISGAFPA
FG08936.1 	SASGSE-ETKRLSLTIPATD--
AN4759.1  	ADAMT-----------------
          	  . :
```
